# Pharmacological and Genetic Suppression of VDAC1 Alleviates the Development of Mitochondrial Dysfunction in Endothelial and Fibroblast Cell Cultures upon Hyperglycemic Conditions

**DOI:** 10.3390/antiox12071459

**Published:** 2023-07-20

**Authors:** Konstantin N. Belosludtsev, Dmitriy A. Serov, Anna I. Ilzorkina, Vlada S. Starinets, Mikhail V. Dubinin, Eugeny Yu. Talanov, Maxim N. Karagyaur, Alexandra L. Primak, Natalia V. Belosludtseva

**Affiliations:** 1 Department of Biochemistry, Cell Biology and Microbiology, Mari State University, pl. Lenina 1, 424001 Yoshkar-Ola, Russia; dubinin1989@gmail.com; 2 Institute of Theoretical and Experimental Biophysics, Russian Academy of Sciences, Institutskaya 3, 142290 Pushchino, Russia; ilzorkinaanna1998@mail.ru (A.I.I.); vlastar@list.ru (V.S.S.); evg-talanov@yandex.ru (E.Y.T.); nata.imagination@gmail.com (N.V.B.); 3 Prokhorov General Physics Institute of the Russian Academy of Sciences, Vavilov St. 38, 119991 Moscow, Russia; dmitriy_serov_91@mail.ru; 4 Institute of Cell Biophysics of the Russian Academy of Sciences, Federal Research Center “Pushchino Scientific Center for Biological Research of the Russian Academy of Sciences”, Institutskaya 3, 142290 Pushchino, Russia; 5 Institute for Regenerative Medicine, Medical Research and Education Center, Lomonosov Moscow State University, 27/10, Lomonosovsky Ave., 119192 Moscow, Russia; m.karagyaur@mail.ru; 6 Faculty of Medicine, Lomonosov Moscow State University, 27/1, Lomonosovsky Ave., 119192 Moscow, Russia; primak.msu@mail.ru

**Keywords:** mitochondria, hyperglycemia, diabetes mellitus, VDAC1, VBIT-4, ROS production

## Abstract

Prolonged hyperglycemia related to diabetes and its complications leads to multiple cellular disorders, the central one being the dysfunction of mitochondria. Voltage-dependent anion channels (VDAC) of the outer mitochondrial membrane control the metabolic, ionic, and energy cross-talk between mitochondria and the rest of the cell and serve as the master regulators of mitochondrial functions. Here, we have investigated the effect of pharmacological suppression of VDAC1 by the newly developed inhibitor of its oligomerization, VBIT-4, in the primary culture of mouse lung endotheliocytes and downregulated expression of VDAC1 in human skin fibroblasts on the progression of mitochondrial dysfunction upon hyperglycemic stress. The cells were grown in high-glucose media (30 mM) for 36 h. In response to hyperglycemia, the mRNA level of VDAC1 increased in endotheliocytes and decreased in human skin fibroblasts. Hyperglycemia induced overproduction of mitochondrial ROS, an increase in the susceptibility of the organelles to mitochondrial permeability transition (MPT) pore opening and a drop in mitochondrial membrane potential, which was accompanied by a decrease in cell viability in both cultures. Treatment of endotheliocytes with 5 µM VBIT-4 abolished the hyperglycemia-induced increase in susceptibility to spontaneous opening of the MPT pore and ROS generation in mitochondria. Silencing of VDAC1 expression in human skin fibroblasts exposed to high glucose led to a less pronounced manifestation of all the signs of damage to mitochondria. Our data identify a mitochondria-related response to pharmacological and genetic suppression of VDAC activity in vascular cells in hyperglycemia and suggest the potential therapeutic value of targeting these channels for the treatment of diabetic vasculopathies.

## 1. Introduction

Glucose is one of the main nutrients used for energy production and storage, and its concentration in plasma is tightly controlled by changes in supply and demand. Metabolic disorders such as diabetes mellitus (DM), which is associated with impaired insulin secretion (type 1 diabetes) or peripheral insulin resistance (type 2 diabetes), can disrupt this control, resulting in a prolonged increase in plasma glucose level or hyperglycemia. Chronic hyperglycemia has been shown to cause severe damage to tissues of the body and life-threatening complications [1]. Today, DM is considered as one of the leading global threats to human health and performance in the 21st century, since about 50% of patients with diabetes are at the most able-bodied age of 40–60 years [2]. Microvascular and macrovascular complications of DM are the most serious manifestations of the disease and the major cause of morbidity and mortality in these patients [2,3,4]. The cells that form the layers of blood vessels, including endothelial cells, vascular smooth muscle cells, and fibroblasts forming adventitia, are at the forefront of exposure to circulating glucose. The abnormalities in vascular cells in diabetes include oxidative stress [5], disturbances of signal transduction [6], changes in nitric oxide (NO) production [7], induction of procoagulant activity [8], increased production of extracellular matrix proteins [9], changes in the expression of adhesion molecules [10], increased permeability, and others [11].

It is generally recognized that at the intracellular level, the dysfunction of mitochondria is closely related to the pathogenesis of DM and hyperglycemic stress [12,13]. Mitochondria are the main cellular organelles responsible for energy production and play a crucial role in the control of cellular homeostasis. They fulfil central roles in redox regulation, maintenance of calcium homeostasis, cell signaling, and apoptosis and serve as a coordinating center for glucose and fatty acid metabolism. Constant dynamic changes in mitochondrial networks via the processes of fusion/fission and turnover of these organelles through mitophagy and mitochondrial biogenesis provide avenues for a cellular response to ever-changing states. Hyperglycemic and diabetic conditions have been found to induce an elevated influx of metabolic substrates into the mitochondria and overload the respiratory chain, resulting in ROS overproduction, an imbalance in ATP synthesis, and a disruption of mitochondrial quality control systems. A growing body of evidence suggests that mitochondria-targeted therapies prevent or delay the occurrence and severity of diabetic conditions [12,14,15,16].

Voltage-dependent anion channels (VDACs) of the outer membrane of mitochondria control the metabolic, ionic, and energy cross-talk between these organelles and the rest of the cell, thus serving as the master regulators of mitochondrial functions. VDACs are permeable to respiratory substrates, small ions, ATP, ADP, and other water-soluble metabolites. Although VDACs are generally recognized to be constitutively open in mitochondria upon aerobic conditions, partial closure of these channels has been suggested to account for the suppression of metabolism in mitochondria in the Warburg phenomenon and inhibition of cell growth [17,18,19,20].

VDACs are a family of pore-forming channel proteins consisting of three isoforms VDAC1-3 encoded by separate genes in humans and mammals. All three isoforms are highly expressed in various tissues and organs, with the prevalence of VDAC1 and VDAC2 over VDAC3 (with the exception in the reproductive tract). VDAC1 is considered to be the most abundant isoform and a key player in mitochondria-mediated apoptosis. Some studies have demonstrated that knockout of VDAC2 is either embryonic lethal or results in severe postnatal defects and developmental delay. The role of VDAC3 has not been fully investigated to date [21,22]. 

Recent studies have indicated that VDAC1 is overexpressed in several diabetic tissues, including pancreatic β-cells and vascular endothelial cells [23,24,25]. As a result, VDAC1 can be mistargeted to the β-cell plasma membrane, which is accompanied by ATP depletion and impaired insulin secretion [23]. Overexpression of VDAC1 is associated with its oligomerization and subsequent formation of a large channel that enables the release of proapoptotic proteins from mitochondria and damage to these organelles due to oxidative stress, calcium dysregulation, and the calcium-dependent formation of the mitochondrial permeability transition (MPT) pore. Therefore, VDAC1 is now considered as a promising therapeutic target to regulate vital metabolic processes and apoptotic cell death [20,26]. Some studies have demonstrated that VDAC blockers can prevent the development of hyperglycemia and maintain normal glucose tolerance in the *db/db* mouse model of type 2 DM [23,27].

The present work aims to study the molecular mechanisms of hyperglycemia-mediated mitochondrial dysfunction and its regulation through pharmacological and genetic suppression of VDAC1 activity in primary endotheliocyte and fibroblast cell cultures. We have examined whether VBIT-4, a novel inhibitor of VDAC activity and oligomerization, is capable of preventing the development of hyperglycemia-induced changes in the mitochondrial membrane potential, ROS generation, and the susceptibility of the organelles to the opening of the MPT pore. In parallel, we have assessed how a decrease in the expression level of VDAC1 in primary human skin fibroblasts affects these alterations in mitochondrial function as well as the gene expression of proteins responsible for mitochondrial biogenesis, mitophagy, and mitochondrial dynamics in the condition of high glucose.

## 2. Materials and Methods

### 2.1. Cell Culture Conditions

Endothelial cells (EC) were isolated from mouse (BALB/c, male, weight 20–22 g) lung microvessels by indirect magnetic separation with rabbit polyclonal anti-CD31-antibodies (Abcam, Cambridge, UK) and magnetic beads conjugated with goat anti-IgG-antibodies (Thermo Fisher, Waltham, MA, USA) [28,29]. Cells were cultured in a DMEM:F12 medium (1:1) supplemented with 10% fetal bovine serum, 100 U/mL penicillin, 2 mM L-glutamine, 100 µg/mL streptomycin, and 50 µg/mL endothelial cell growth supplement according to the standard protocols [28,29]. The culture of EC was obtained by combining cells isolated from three mice. Round 25 mm coverslips were placed one at a time into the wells of 6-well gelatinized culture plates (0.2% gelatin). Then, a suspension of EC in the culture medium was added. Cells were cultured for three days until confluence level of 90% or more was reached. Cultured cells between passages 7 and 10 were used in the experiments.

Primary human dermal fibroblasts (HF) derived from a patch of skin of healthy donors (*n* = 3) were obtained from the biobank of the Institute for Regenerative Medicine, Lomonosov Moscow State University, collection ID: MSU_FB (https://human.depo.msu.ru, accessed on 18 July 2023). Cells were cultured in DMEM containing 10% fetal bovine serum and 1% of Antibiotic Antimycotic (Pen/Strep/Fungizone) solution. Cells were used between the 10th and 13th passages.

### 2.2. CRISPR/Cas9-Mediated Knockdown of VDAC1 Gene in Primary Human Fibroblasts

A pair of LentiCRISPRv2GFP (Addgene, Watertown, MA, USA #82416) constructs, modified to express Cas9-D10A nickase variant, was used to knockout VDAC1 gene (NC_000005.10 Chromosome 5 Reference GRCh38.p14 Primary Assembly: VDAC1 (Gene ID:7416, GenBank: NP_003365.1) https://www.ncbi.nlm.nih.gov/gene/7416, accessed on 18 July 2023) in primary human fibroblasts. gRNA protospacers were designed and cloned in LentiCRISPRv2GFP-D10A_nickase using BsmBI sites as described earlier [30,31]. The specificity of gRNA protospacers was assessed using COSMID: CRISPR Search with Mismatches, Insertions and/or Deletions [32]. Obtained protospacers are listed in Table 1. Lentiviral particles encoding components of a CRISPR/Cas9 genome editing system were compiled as outlined earlier [33]. HF transduction was performed as described earlier [34]. Lentiviral constructs, bearing VDAC1-gRNA1 and VDAC1-gRNA2, as well as VDAC1-gRNA3 and VDAC1-gRNA4, were used in pairs. Transduced cells with the most intense fluorescence in GFP-channel were selected (using a BD FACSAria III cell sorter) and cloned. To confirm VDAC1 knockout, genomic DNA was isolated from the obtained clones and amplified using the primers listed in Table 1. Amplicons were sequenced by DNA sequencing by Sanger’s method. The data were processed using the Chromas 2.6.6 software (Technelysium Pty Ltd., Brisbane, Australia). The design and sequencing results of CRISPR/Cas9-mediated editing of *VDAC1* gene in primary human fibroblasts are demonstrated in Figure S1. The “TIDE: Tracking of Indels by the Decomposition” software [35] was used for analysis of sequencing results of the edited genes to elucidate the effectiveness of genome editing. Individual clones of primary human fibroblasts, in which the efficiency of *vdac1* editing was higher than 65%, were used in the experiments.

### 2.3. Hyperglycemia Induction

To induce hyperglycemic stress, the cells were exposed to a high concentration of D-glucose (30 mM) for 36 h in a CO_2_ incubator (Sanyo, Osaka, Japan). Control cells were grown for 36 h in a culture medium with a glucose concentration of 5 mM. VDAC1-interacting molecule VBIT-4 was added to the culture medium as a solution in DMSO (1:2000 dilution). In control experiments (without VBIT-4), the cells were incubated for 36 h after the addition of DMSO in the appropriate volume.

### 2.4. Measurement of Cell Viability

To evaluate cell viability, the cells were washed three times with Hank’s balanced salt solution and incubated with 5 µM propidium iodide (λex = 493 nm, λem 636 nm) and 5 µg/mL of Hoechst 33342 vital dye (λex = 361 nm, λem 497 nm) at 37 °C. Fluorescence levels were detected using an LED imaging system based on an AE31E inverted microscope (Motic, Barselona, Spain) [28]. Analysis of the images obtained was performed using the Image J2 (Fiji) software version 2.3.1 (NIH, Bethesda, MD, USA). In each sample 200–400 cells were analyzed.

### 2.5. Measurements of the Mitochondrial Membrane Potential, ROS Production, and MPT Pore Opening in Cells

To evaluate the mitochondrial membrane potential, the cells were co-incubated with the fluorescent cationic indicator rhodamine 123 (λex = 485 nm, λem 525 nm) and then treated with the mitochondrial uncoupling agent FCCP (2 µM). Fluorescence intensity of rhodamine 123 was presented as the ratio of fluorescence intensity signal at each point (F) to average fluorescence intensity signal under basic conditions (F_0_). Change in the fluorescence intensity in the control cells was taken as 100% [28]. 

ROS generation was measured using the fluorescent dye 2′,7′-dichlorodihydrofluorescein diacetate (H_2_DCFDA) (λex = 490 nm, λem 520 nm). Cells of the four experimental groups were co-incubated with 20 µM H_2_DCFDA for 30 min at 37 °C [28], and 1 µM H_2_O_2_ was used as a positive control in these experiments. In each sample, 200–400 cells were analyzed. 

Fluorescence signals of H_2_DCFDA and rhodamine 123 were detected using an LED imaging system based on an AE31E inverted microscope (Motic, Barselona, Spain), and further data analysis was performed using the Image J2 (Fiji) software version 2.3.1 (NIH, Bethesda, MD, USA).

Assessment of the MPT pore opening in cells was carried out using the fluorescence probe calcein acetoxymethyl ester (calcein-AM, λex = 494 nm, λem 517 nm) in the presence of CoCl_2_ according to standard protocols described in [24,28]. Briefly, cultured cells were washed with Hank’s balanced salt solution and incubated for 30 min at 37 °C in the presence of 200 nM MitoTracker Red (to visualize the mitochondrial structure), 1 µM calcein-AM, and 1 mM CoCl_2_ (to quench cytosolic calcein-AM fluorescence). After staining, the cells were washed with Hank’s solution, and fluorescence intensity was measured by a DMI6000 confocal microscope (Leica Microsystems, Wetzlar, Germany).

### 2.6. Electrophoresis and Immunoblotting

Cells were washed twice with cold PBS and then treated with 2× loading buffer containing 4% SDS, 20% glycerol, 100 mM Tris-HCl (pH 6.8). The resulting suspension was incubated for 10 min at 95 °C, resuspended by pipetting, and centrifuged at 10,000× *g* for 5 min. The supernatant was transferred to a new Eppendorf tube and used to determine the protein concentration using the Quick Start™ Bradford Protein Assay Kit. The samples were diluted in Laemmli buffer, run on 12.5% SDS–PAGE (10 μg/lane), and transferred to a 0.45 μm nitrocellulose membrane (Cytiva, Marlborough, MA, USA). Then, the membrane was blocked with PBS/3% nonfat dry milk overnight at 4 °C and incubated with the appropriate primary antibody. The anti-VDAC1 (ab15895), anti-GAPDH (ab181602) were from Abcam. Enhanced chemiluminescence detection reagents (Pierce, Rockford, IL, USA) were applied to measure the peroxidase activity. Proteins were analyzed using the LI-COR system (LI-COR, Lincoln, NE, USA) (Figure S2). Optical density data were recorded on the LI-COR Image Studio software.

### 2.7. Quantitative Real-Time PCR

To calculate the level of expression of genes encoding the proteins responsible for mitophagy, mitochondrial biogenesis, and mitochondrial fission/fusion, real-time quantitative PCR was carried out using a QuantStudio 1 amplifier (Thermo Fisher Scientific, Waltham, MA, USA). Selection and analysis of the gene-specific primers was conducted using Primer-BLAST [36]. The sequence of oligonucleotides is presented below (Table 2). Total RNA was extracted from approximately 1 × 10^7^ cells per sample using an ExtractRNA reagent (Eurogen, Moscow, Russia). The differences between experimental and the control values were calculated according to the formula ∆∆Ct = ∆Ct (Control) − ∆Ct (experiment); each ∆Ct value was estimated using the formula ∆Ct = Ct (tested gene) − Ct (Rplp2) [37].

### 2.8. Statistical Data Processing

Statistical data analysis was conducted using the GraphPad Prism version 8.4 software (GraphPad Software Inc., San Diego, CA, USA). The data were presented as mean ± standard deviation (*n* = 4–6, where *n* is the number of independent experiments with different cell cultures). To verify that the data had Gaussian distribution, we used the Shapiro–Wilk normality test. For normally distributed data, one-way analysis of variance (ANOVA) with post hoc Tukey multiple comparison test was applied.

## 3. Results

### 3.1. Hyperglycemia Causes an Increase in VDAC1 Gene Expression in Primary Lung Endothelial Cells but a Decrease in Its Expression in Primary Fibroblasts

**Figure 1:**
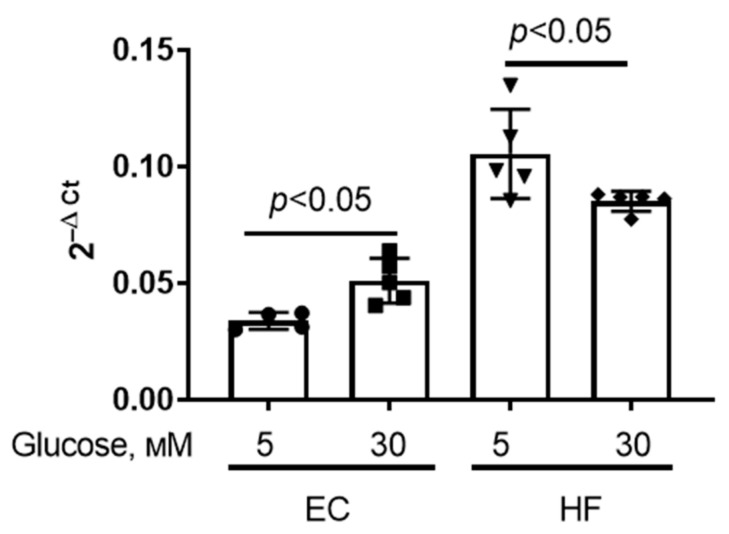
*Vdac1* mRNA level in primary mouse microvascular endothelial cells (EC) and human skin fibroblasts (HF) under normal (5 mM) and high (30 mM) glucose conditions. Means ± SD are shown (*n* = 4–5).

As mentioned above, the development of diabetes mellitus is accompanied by an increase in VDAC1 expression in a number of tissues and organs [23,24]. Therefore, we evaluated the effect of hyperglycemia on changes in *Vdac1* gene expression in primary human skin fibroblast cells and mouse microvascular endothelial cells. Cells were exposed to high glucose (30 mM) for 36 h to induce hyperglycemic stress (control cells incubated in medium containing 5 mM glucose). One can see that hyperglycemic conditions caused an increase in *Vdac1* mRNA level in endothelial cells but its decrease in fibroblasts (Figure 1).

### 3.2. VBIT-4 Prevents the Development of Cell Death and Dysfunction of Mitochondria in Primary Lung Endothelial Cells upon Hyperglycemic Stress

**Figure 2:**
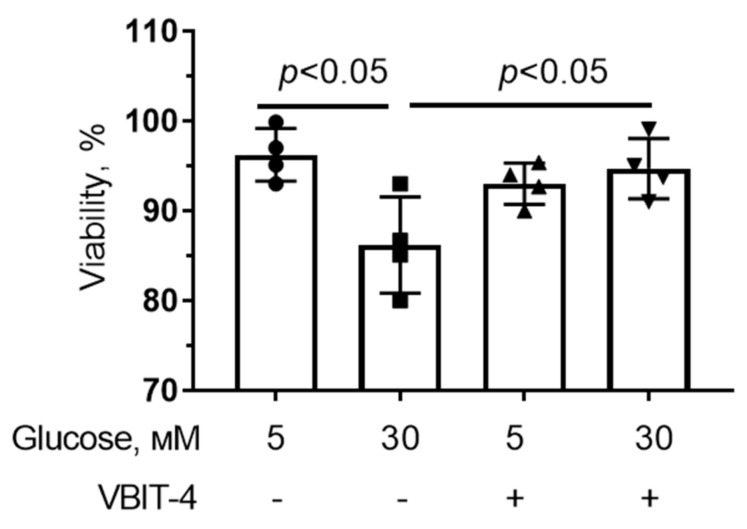
Effect of VBIT-4 (5 µM) on the viability of mouse microvascular endothelial cells under normoglycemia (5 mM glucose) and high (30 mM) glucose conditions. Means ± SD are shown (*n* = 4).

Figure 2 shows that hyperglycemia resulted in a small but significant decrease in cell viability. Incubation of cells with VBIT-4 (5 μM) (VDAC inhibitor [38]) prevented the decrease in the viability of endothelial cells under conditions of hyperglycemia.

**Figure 3:**
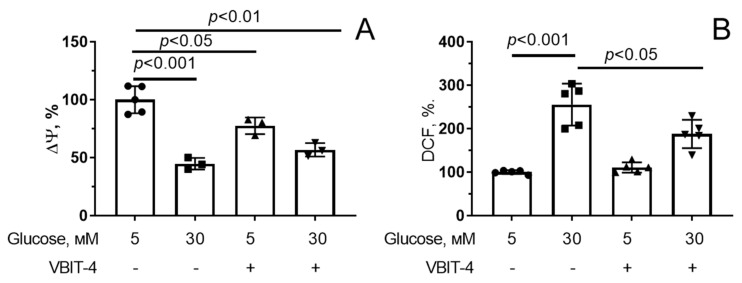
Effect of VBIT-4 (5 µM) on the mitochondrial membrane potential (Δψ) (**A**) and production of reactive oxygen species (**B**) in mouse microvascular endothelial cells under normo- (5 mM glucose) and hyperglycemia (30 mM glucose). Means ± SD are shown (*n* = 3–5).

It was found that VBIT-4 could directly interact with VDAC1, reduce channel activity and decrease the permeability of the outer mitochondrial membrane by preventing VDAC oligomerization [19,38]. Here, we evaluated the effect of the inhibitor of mitochondrial VDACs, VBIT-4, on the development of mitochondrial dysfunction in cells under conditions of hyperglycemia. As shown in Figure 3A and Figure S2, hyperglycemia significantly decreased the mitochondrial membrane potential in endothelial cells. It should be noted that 5 μM VBIT-4 alone reduced the membrane potential of the organelles under normoglycemic conditions. However, it did not affect the mitochondrial membrane potential under conditions of hyperglycemia.

In parallel with mitochondrial depolarization, there was a more than twofold increase in ROS generation in endothelial cells exposed to hyperglycemia (Figure 3B and Figure S3). One can see that 5 µM VBIT-4 suppressed hyperglycemia-induced H_2_O_2_ overproduction in endothelial cells.

**Figure 4:**
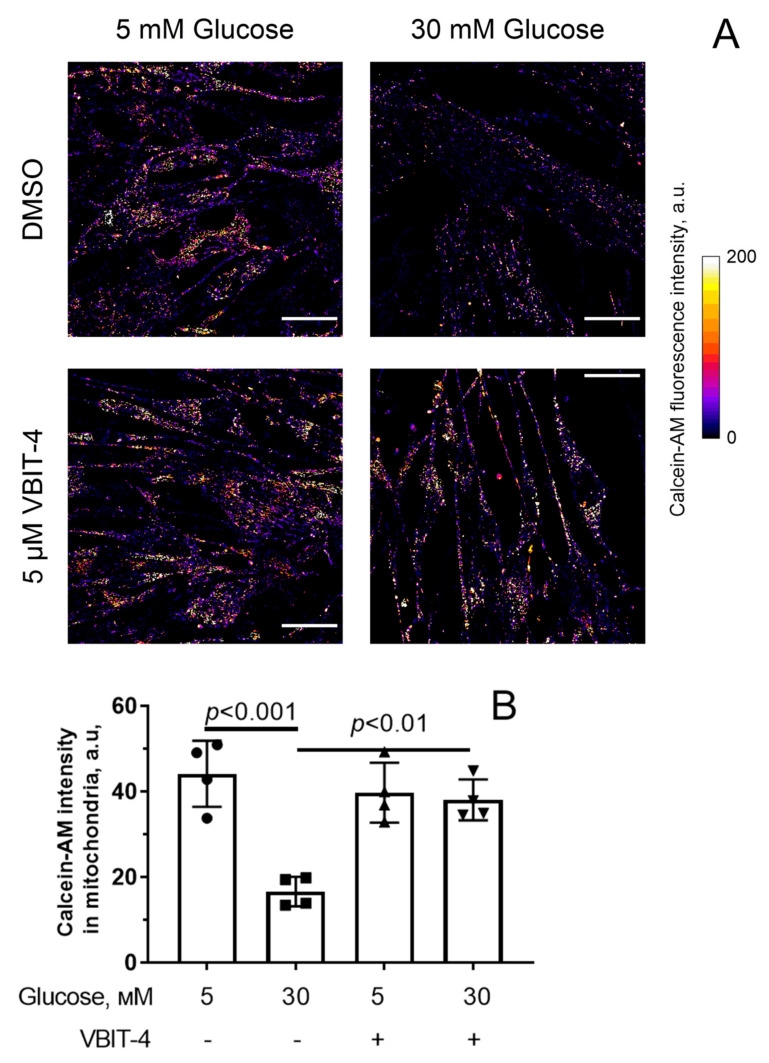
MPT pore opening in mouse microvascular endothelial cells. (**A**) Representative fluorescence images of calcein-AM in the presence of CoCl_2_ in endothelial cells of the experimental groups. Scale bar—25 μm. (**B**) Intensity of calcein-AM fluorescence signals in mitochondria of the microvascular endothelial cells from four experimental groups. Means ± SD are shown (*n* = 4).

One of the manifestations of mitochondrial dysfunction is the opening of the MPT pore in the inner mitochondrial membrane [39]. We assessed the spontaneous formation of the MPT pore in mitochondria using calcein-AM fluorescence in the presence of cobalt ions. The MitoTracker DeepRed was used to determine the mitochondrial localization of calcein-AM. Figure 4 shows that the intensity of calcein fluorescence signals in mitochondria of endothelial cells incubated in 30 mM glucose for 36 h in the presence of cobalt ions significantly decreased, indicating the release of calcein-AM from the mitochondria due to the MPT pore opening. We have previously demonstrated that the highly selective mitochondrial pore inhibitor alisporivir significantly increased the level of calcein fluorescence in endotheliocyte mitochondria under hyperglycemic conditions [28]. This suggests that hyperglycemia is associated with increased spontaneous induction of the MPT pore. Pre-incubation of endotheliocytes with 5 µM VBIT-4 under hyperglycemic conditions resulted in a significant increase in calcein fluorescence in mitochondria. Thus, although 5 μM VBIT-4 itself reduced the membrane potential of mitochondria, it was able to prevent the MPT pore induction under hyperglycemic conditions.

### 3.3. Suppression of VDAC1 Expression in Human Skin Fibroblasts Normalizes Mitochondrial Function in Hyperglycemia

**Figure 5:**
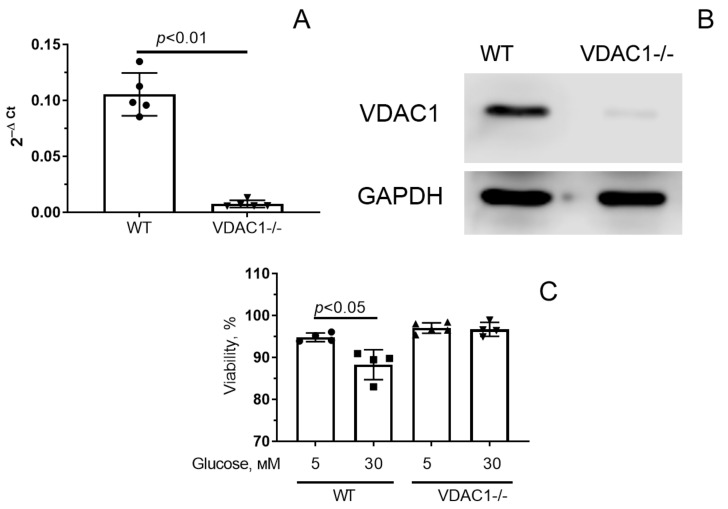
mRNA level (**A**) and amount of VDAC1 protein (**B**) in cells with normal (WT) and decreased (VDAC1-/-) expression of VDAC1. (**C**) Survival of fibroblasts with normal and reduced expression of VDAC1 under normal (5 mM) and high (30 mM) glucose conditions (**C**). Means ± SD are shown (*n* = 4–5).

As follows from the results obtained, the pharmacological suppression of VDACs with VBIT-4 led to the mitigation of mitochondrial dysfunction in hyperglycemia. In the next part of the work, we investigated how the decrease in VDAC1 gene expression in primary human skin fibroblasts affects hyperglycemia-induced mitochondrial damage. Using the CRISPR/Cas9-mediated genome editing technique, we obtained individual clones of primary human fibroblasts in which the protein and mRNA levels of VDAC1 were decreased by approximately 90% (Figure 5A,B). It is important to note that the cells with reduced (VDAC1-/-) expression of VDAC1 had a larger size (area parameter) compared to wild-type cells (616 ± 12 vs. 551 ± 11 μm^2^, *p* < 0.01).

Figure 5C shows the viability of control fibroblasts and VDAC1 down-expressing fibroblasts under normo- (5 mM glucose) and hyperglycemic (30 mM glucose) conditions. One can see that hyperglycemia is accompanied by a decrease in the viability of control human skin fibroblasts. At the same time, cells with reduced VDAC1 expression were practically insensitive to an increase in the level of glucose in the medium. In this case, the percentage of living cells in both glucose concentrations was approximately equal.

**Figure 6:**
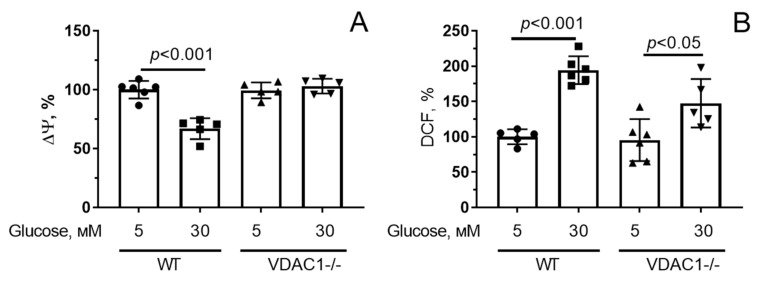
Changes in the mitochondrial membrane potential (Δψ) (**A**) and production of reactive oxygen species (**B**) in human skin fibroblasts with normal (WT) and reduced (VDAC1-/-) expression of VDAC1 under conditions of normo- (5 mM glucose) and hyperglycemia (30 mM glucose). Means ± SD are shown (*n* = 3–5).

As in the case of microvascular endothelial cells, exposure of primary human skin fibroblasts to hyperglycemia caused depolarization of the inner mitochondrial membrane (Figure 6A and Figure S4) and hyperproduction of H_2_O_2_ (approximately two-fold compared with normoglycemia) (Figure 6B and Figure S5). Under hyperglycemic stress, cells with reduced VDAC1 expression did not show mitochondrial depolarization, and H_2_O_2_ production increased by 1.6 times compared to normoglycemia.

**Figure 7:**
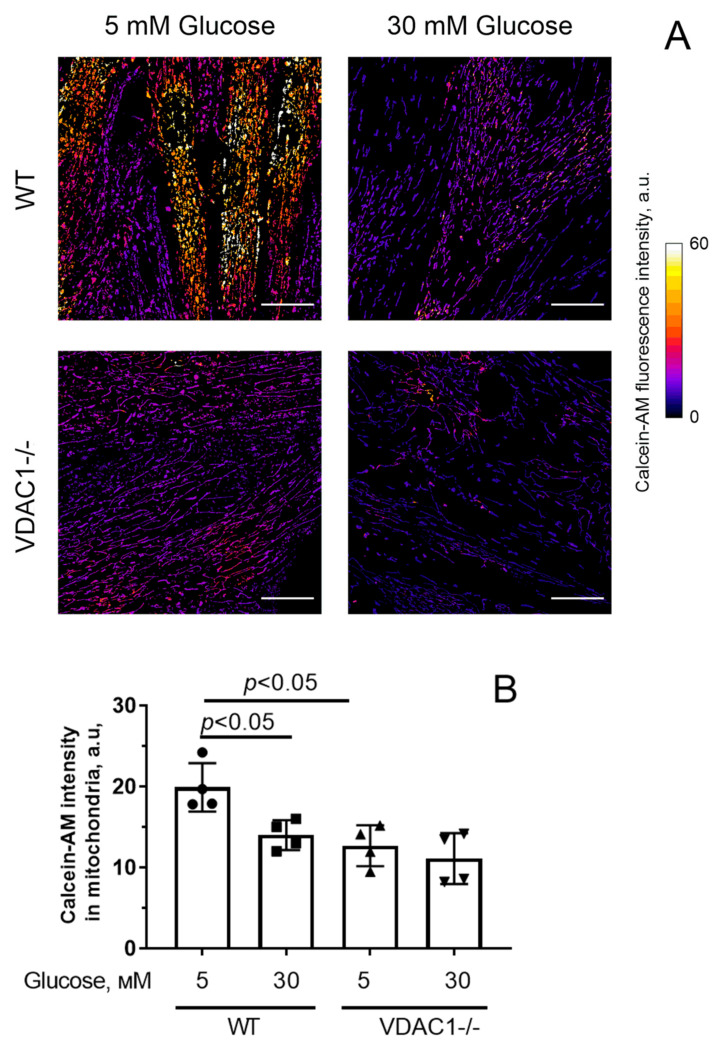
MPT pore opening in human skin fibroblasts with normal (WT) and reduced (VDAC1-/-) expression of VDAC1. (**A**) Typical fluorescence images of mitochondrial calcein in the presence of CoCl_2_ in fibroblasts of the experimental groups. Scale bar—25 μm. (**B**) Intensity of calcein fluorescence in fibroblasts mitochondria from four experimental groups. Means ± SD are shown (*n* = 4).

Figure 7 shows spontaneous MPT pore activity data. Similar to the picture observed in the culture of mouse lung endothelial cells, hyperglycemia caused quenching of calcein-AM fluorescence in the presence of cobalt ions (1.4-fold). At the same time, the relative decrease in calcein fluorescence induced by hyperglycemia in cells with reduced VDAC1 expression was less pronounced (1.18-fold). All this may indicate that the decrease in VDAC1 expression may be a protective mechanism under conditions of hyperglycemia.

**Figure 8:**
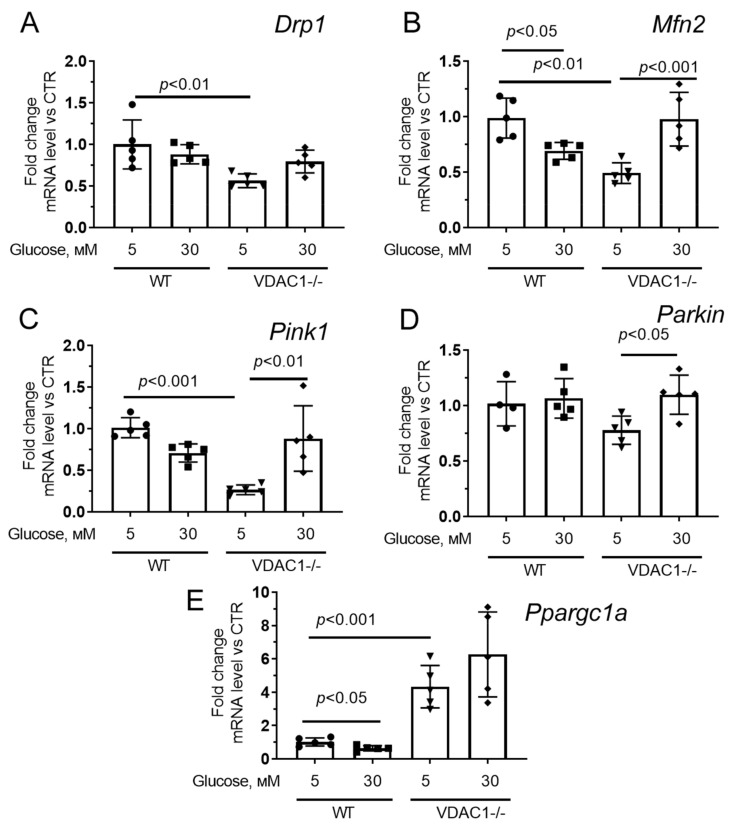
The relative mRNA levels of *Drp1* (**A**), *Mfn2* (**B**), *Pink1* (**C**), *Parkin* (**D**), and *Ppargc1a* (**E**) in the human skin fibroblasts in the experimental groups. Means ± SD are shown (*n* = 5).

We also assessed the expression of genes encoding the proteins responsible for mitochondrial dynamics, biogenesis, and mitophagy (Figure 8). One can see that hyperglycemia caused a significant decrease in *Mfn2* expression in human skin fibroblasts, suggesting a decline in the amount of mitofusin2 involved in mitochondrial fusion. In parallel, there was a decrease in the mRNA level of the *Ppargc1a* gene responsible for the synthesis of PGC1a, a molecule involved in the processes of mitochondrial biogenesis. The expression of the *Pink1* and *Parkin* genes responsible for the synthesis of proteins involved in mitophagy, as well as the *Drp1* gene responsible for the synthesis of the mitochondrial fission protein, did not change significantly.

Fibroblasts with reduced expression of VDAC1 under normoglycemic conditions showed a significant decrease in the expression of *Drp1*, *Mfn2*, *Pink1* (but not *Parkin*) genes compared to control fibroblasts. Hyperglycemia exhibited a significant increase in the expression of these genes to the level of normoglycemia in control cells. One should note that we observed a significant increase in the expression of the *Ppargc1a* gene both under conditions of normoglycemia and hyperglycemia. 

## 4. Discussion

Mitochondrial dysfunction is a key cellular event in hyperglycemia and diabetes mellitus. In this regard, the regulation of mitochondrial homeostasis is one of the tools for the treatment of this disease. Many studies have shown that pharmacological or genetic modulation of the activity of various mitochondrial proteins not only contributes to the normalization of mitochondrial function but also alleviates the consequences of diabetes mellitus [12,14,40,41,42,43].

Mitochondrial outer membrane voltage-dependent anion channels (VDACs) are the main routes for the exchange of metabolites and ions between mitochondria and the cell cytoplasm. A number of studies have shown that the development of diabetes mellitus is accompanied by an increase in VDAC1 expression, which may be one of the factors in the development of this pathology [23,24]. In particular, it was found that hyperglycemia in pancreatic beta cells leads to erroneous translocation of this protein into the plasma membrane of the cell, a significant decrease in the ATP pool, and impaired insulin secretion [23]. Increased expression of VDAC1 has been suggested to be the cause of apoptotic death of mouse coronary endothelial cells isolated from diabetic mice [24]. In this regard, in this work, we focused on evaluating the effect of pharmacological or genetic suppression of VDAC1 activity on the development of mitochondrial dysfunction in cell culture, which is observed in response to hyperglycemic stress.

As shown in this work, hyperglycemia (30 mM glucose for 36 h) leads to a small but significant decrease in the survival of both mouse lung endothelial cells and human skin fibroblasts. This decrease in survival may be based on the development of mitochondrial dysfunction, whose main events are ROS overproduction, an increase in the spontaneous activity of the MPT pore, and, as a result, a drop in the membrane potential. It is important to note that these two cell cultures differ in their response in terms of VDAC1 expression to hyperglycemia. Hyperglycemia induced an increase in the expression of the *Vdac1* gene in endothelial cells, while fibroblasts showed a decrease in the *Vdac1* mRNA level. Such tissue specificity has been described previously in a number of works [23,24,44,45,46,47]. Moreover, it was previously shown that mitochondria of human and animal tissue cells react differently to the development of diabetes mellitus [12,48]. In particular, it was found that the mitochondria of the skeletal and cardiac muscles become more sensitive to the induction of the MPT pore during the development of diabetes mellitus, while the liver mitochondria become more resistant [12,40,49,50]. Another example is that in most cells of the human and animal body, diabetes mellitus leads to a decrease in mitochondrial biogenesis, while an increase in PGC1a expression is observed in liver cells [12]. Thus, it cannot be rigorously stated that an increase in the level of VDAC1 (and/or other proteins) can directly underlie the pathological changes in the cell observed in diabetes/hyperglycemia.

At the same time, according to our results, a change in channel activity (suppression of VDAC oligomerization) or a significant decrease in the expression level of VDAC1 contributes to the normalization of mitochondrial function and prevents cell death during hyperglycemia. We used VBIT-4, a novel VDAC inhibitor, as a pharmacological modulator. This compound has previously been shown to prevent the development of hyperglycemia in *db/db* mice at sufficiently high concentrations [23]. As follows from the data obtained in the work, 5 μM VBIT-4 prevents the death of cells in the primary culture of mouse lung endothelial cells and partially normalizes the functional activity of mitochondria. In particular, VBIT-4 normalized the generation of H_2_O_2_ in the cell and decreased the spontaneous activity of the MPT pore opening. These positive effects of the agent may be due to the fact that VBIT-4 is able to reduce the formation of oligomeric forms of VDAC1 [38,51]. Recent studies demonstrated that VDAC1 oligomerization causes an increase in the permeability of the outer mitochondrial membrane, which can lead to the induction of inflammation and apoptosis in various cell lines [51]. At the same time, VBIT-4 had practically no effect on mitochondrial depolarization induced by hyperglycemia. Moreover, under conditions of normoglycemia, VBIT-4 itself caused a drop in the membrane potential of mitochondria in endothelial cells. A similar effect of this VDAC1-interacting molecule was also observed when using primary human skin fibroblasts under conditions of normoglycemia (in press). Additional studies performed on isolated rat liver mitochondria showed that VBIT-4 decreased the mitochondrial membrane potential in a dose-dependent manner (Figure S6). This suggests that this agent is an uncoupler of oxidative phosphorylation. The revealed effect can lead to the stimulation of intracellular energy metabolism and, thereby, the normalization of cell vital activity under conditions of hyperglycemia.

A significant (10-fold) decrease in VDAC1 expression in human skin fibroblasts also leads to suppression of the consequences of hyperglycemic stress. Under conditions of hyperglycemia, cells with reduced expression of VDAC1 demonstrate less dysfunctional changes in the mitochondrial membrane potential, H_2_O_2_ generation rate, and spontaneous activity of MPT pore opening compared to control fibroblasts. Moreover, cells with suppressed expression of VDAC1 practically did not lose their viability under conditions of hyperglycemia. VDAC1 is known to be a proapoptotic protein [19,25]. A decrease in its expression may explain this effect.

At the same time, it should be noted that cells with reduced expression of VDAC1 under normoglycemic conditions show a significant decrease in the expression of genes responsible for mitochondrial dynamics and mitophagy. In the case of hyperglycemia, the expression of these genes reached the values observed in control fibroblasts under normoglycemia. However, the expression of *Ppargc1a*, which is responsible for the synthesis of the mitochondrial biogenesis regulator PGC-1α is significantly increased in these cells, regardless of the glucose concentration. This suggests that reduced expression of VDAC1 in the cell contributes to the activation of compensatory mechanisms for increased glucose utilization, which may include, for example, an increase in mitochondrial mass. Switching the cell to a high sugar level contributes to its stabilization and an increase in the activity of not only mitochondrial biogenesis but also other processes responsible for mitochondrial homeostasis. Altogether, our findings indicate that VDAC1 depletion affects the morphology of the mitochondrial network in cells in both normal and high glucose levels. Therefore, further studies are needed to evaluate how downregulation of VDAC1 affects the ultrastructure and functional activity of mitochondria in various cells in response to ever-changing metabolic conditions. It will also be important to assess possible alterations in the protein level of the molecules responsible for mitochondrial biogenesis, dynamics, and mitophagy in order to establish these mechanisms in more detail.

In addition to the above, this study contains several other limitations. Although VBIT-4 treatment has already shown a protective effect against hyperglycemia-induced mitochondrial dysfunction in cultured cells and the progression of diabetes mellitus in mice, its effect on mitochondria, according to our data, is rather controversial. To further elucidate the therapeutic potential of pharmacological modulation of VDAC1, it is necessary to investigate how other VDAC blockers affect the progression of diabetic hyperglycemia. It is also necessary to determine how a decrease in VDAC1, as a vital metabolic enzyme, affects the functioning of various cells in normal and diabetic conditions. All of these studies could contribute to the development of a novel strategy based on mitochondria-targeted modulation for the treatment of diabetes and its complications.

## 5. Conclusions

The results of this work demonstrate that: (1) under conditions of hyperglycemia, the level of VDAC1 mRNA increases in endothelial cells and decreases in fibroblasts; (2) hyperglycemia causes the development of mitochondrial dysfunction and decreases cell viability in the primary cultures of mouse microvascular endothelial cells and human skin fibroblasts; (3) VBIT-4, an inhibitor of VDAC activity and oligomerization, alleviates the development of hyperglycemia-induced mitochondrial dysfunction in the cells; and (4) downregulated VDAC1 expression in cultured primary fibroblasts leads to a less pronounced manifestation of signs of mitochondrial damage in hyperglycemia. All the findings indicate that hyperglycemic stress induces similar effects in the primary cells of different origins, and genetic or pharmacological modulation of VDAC1 in the cells suppressed the negative effects of high glucose on mitochondrial function. Taken together, this suggests that reducing VDAC1 activity may be a promising strategy for the treatment of diabetic hyperglycemia and its complications.

## Figures and Tables

**Table 1:** Nucleotide sequences and characteristics of gRNA protospacers and primers used for genome editing and amplification of the target DNA site.

Name	Sequence	Amplicon Length, bp	T_melting_, °C
VDAC1-gRNA1	TTTTCTGTTCAGCTTGCACG	-	-
VDAC1-gRNA2	TGCCACTAGATTTAGTCACA		
VDAC1-gRNA3	TTCTCTGATGTTGCAGGTGG	-	-
VDAC1-gRNA4	TTACCCAGTGTTAGGTGAGA		
VDAC1-test-f	AGGGTCTTGCCTCTTGCAGAAA	725	59
VDAC1-test-r	AGCTCCTTGGCGGGTAACAA		

**Table 2:** List of gene-specific primers for RT-PCR analysis.

Gene	Forward (5′→3′)	Reverse (5′→3′)
Human gene-specific primer		
*Pink1*	AGCCACCATGCCTACATTGC	TGGAGGAACCTGCCGAGATG
*Prkn*	ACAGCAGGAAGGACTCACCA	TGCTGCACTGTACCCTGAGT
*D* *rp1*	CTTCGGAGCTATGCGGTGGT	GCAGGACGAGGACCAGTAGC
*Mfn2*	AAGTGGAGAGGCAGGTGTCG	TCCTCTATGTGGCGGTGCAG
*Ppargc1a*	GCCTTCCAACTCCCTCATGG	CTCCGGAAGAAACCCTTGCAT
Vdac1	CGCCTGCTTCTCGGCTAAAG	CCAGCATTGACGTTCTTGCC
*Rplp2*	GACGACCGGCTCAACAAGGT	CCAATACCCTGGGCAATGACG
Mouse gene-specific primer		
*Vdac1*	AGTGACCCAGAGCAACTTCGCA	CAGGCGAGATTGACAGCAGTCT
*Rplp2*	CGGCTCAACAAGGTCATCAGTGA	AGCAGAAACAGCCACAGCCCCAC

## Data Availability

The data presented in this study are available upon request from the corresponding author.

## Supplementary Materials

The following supporting information can be downloaded at: https://www.mdpi.com/article/10.3390/antiox12071459/s1, Figure S1: Design of gRNA protospacers to the VDAC1 gene (A) and results of analysis of its nucleotide sequence after genome editing (B, C) within the culture of primary human fibroblasts. In panel A: gRNA protospacers are underlined with the red line, the corresponding PAM sequences are marked with red boxes, and the cut sites are marked with red wedges. B and C—analysis of the complex of genomic variants obtained after VDAC1-gene editing in individual clones of primary fibroblasts: top—the results of analysis with “TIDE: Tracking of Indels by DEcomposition” software; bottom—the summation of the edited VDAC1 alleles visualized with Chromas 2.6.6 software; Figure S2: Representative images of the intensity of rhodamine 123 fluorescence signals in the primary culture of mouse microvascular endothelial cells from four experimental groups before and after the addition of the uncoupler of oxidative phosphorylation carbonylcyanide-p-trifluoromethoxyphenylhydrazone (FCCP, 2 µm). The scale bar is 100 μm; Figure S3: Representative images of the intensity of 2′,7′-dichlorodihydrofluorescein diacetate (H2DCFDA) fluorescence signals in the primary culture of mouse microvascular endothelial cells from four experimental groups: (1) 5 mM glucose + 0.1% DMSO; (2) 5 mM glucose + 5 μM VBIT-4; (3) 30 mM glucose + 0.1% DMSO; (4) 30 mM glucose + 5 μM VBIT-4. The scale bar is 100 μm; Figure S4: Typical images of the intensity of rhodamine 123 fluorescence signals in the culture of primary human skin fibroblasts with normal (WT) and reduced (VDAC1-/-) expression of VDAC1 under conditions of normo- (5 mM glucose) and hyperglycemia (30 mM glucose) before and after the addition of the uncoupler of oxidative phosphorylation carbonylcyanide-p-trifluoromethoxyphenylhydrazone (FCCP, 2 µm). The scale bar is 100 μm; Figure S5: Typical images of the intensity of 2′,7′-dichlorodihydrofluorescein diacetate (H2DCFDA) fluorescence signals in the culture of primary human skin fibroblasts with normal (WT) and reduced (VDAC1-/-) expression of VDAC1 under conditions of normo- (5 mM glucose) and hyperglycemia (30 mM glucose). The scale bar is 100 μm; Figure S6: Representative recordings of changes in the fluorescence intensity of safranine O which reveal VBIT-4-induced depolarization of isolated rat liver mitochondria energized by the complex I substrates (2.5 mM potassium glutamate + 2.5 mM malate). Additions: 15 µM VBIT-4, 15 µM VBIT-4, 50 µM DNP. Typical traces are shown (*n* = 5).
