# Supplementary material for: Pharmacological and Genetic Suppression of VDAC1 Alleviates the Development of Mitochondrial Dysfunction in Endothelial and Fibroblast Cell Cultures upon Hyperglycemic Conditions
Source: Antioxidants (Basel). 2023 Jul 20;12(7):1459. doi: 10.3390/antiox12071459 (PMC10376467; doi:10.3390/antiox12071459)
Supplement: Supplementary file 1 [file antioxidants-12-01459-s001.zip › antioxidants-2503420-supplementary.pdf]

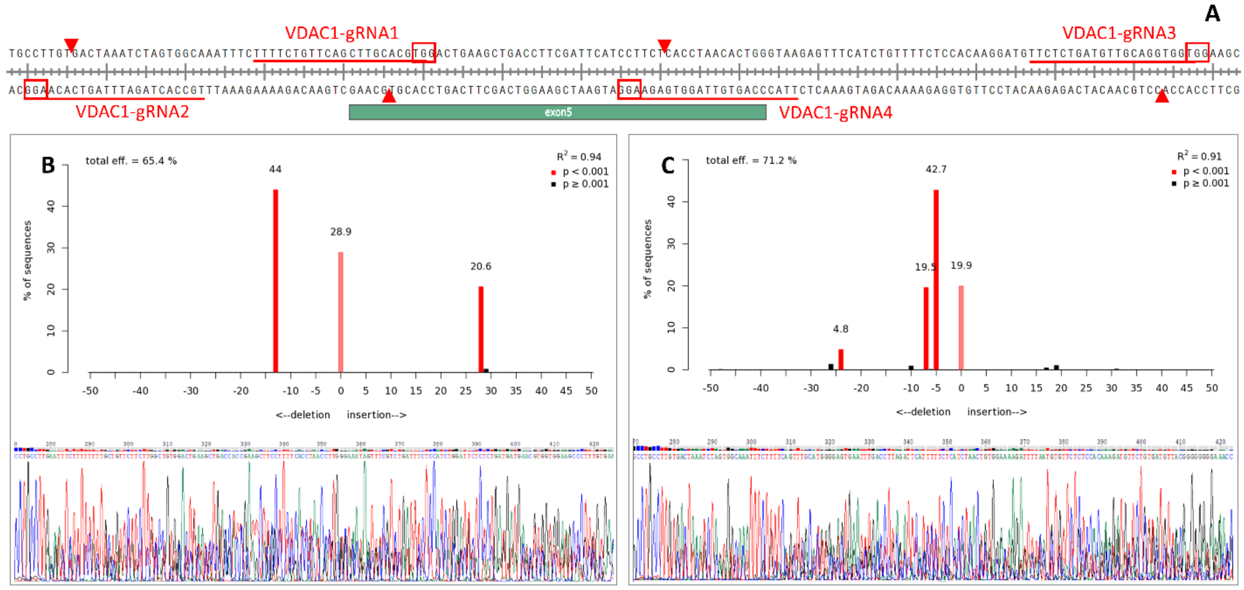

Figure S1. Design of gRNA protospacers to the VDAC1 gene (A) and results of analysis of its nucleotide sequence after genome editing (B,C) within the culture of primary human fibroblasts. In panel A: gRNA protospacers are underlined with the red line, the corresponding PAM sequences are marked with red boxes and the cut sites are marked with red wedges. B and C - Analysis of the complex of genomic variants obtained after VDAC1-gene editing in individual clones of primary human fibroblasts: top - the results of analysis with the "TIDE: Tracking of Indels by DEcomposition" software; bottom - the summation of the edited VDAC1 alleles visualized with the Chromas 2.6.6 software.

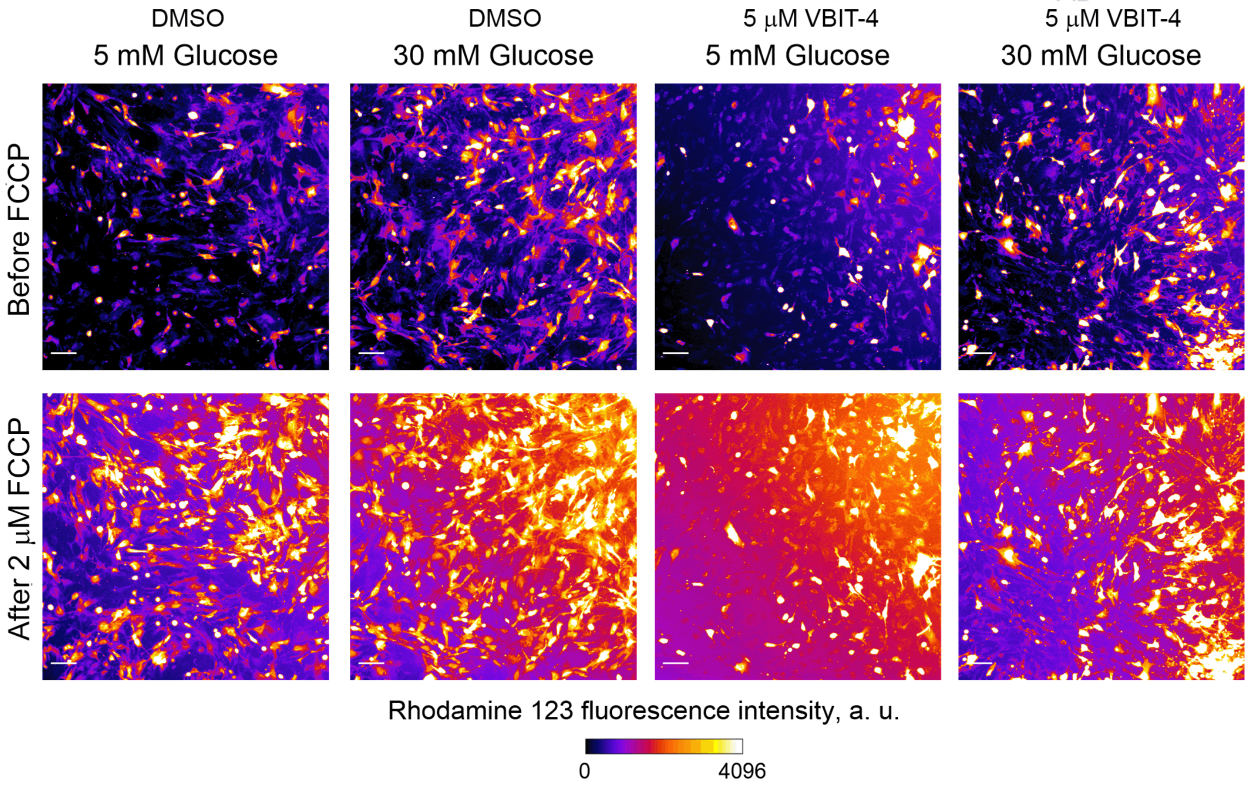

Figure S2. Representative images of the intensity of rhodamine 123 fluorescence signals in the primary culture of mouse microvascular endothelial cells from four experimental groups before and after the addition of the uncoupler of oxidative phosphorylation carbonylcyanide-p-trifluoromethoxyphenylhydrazone (FCCP, 2 μm). The scale bar is 100 μm.

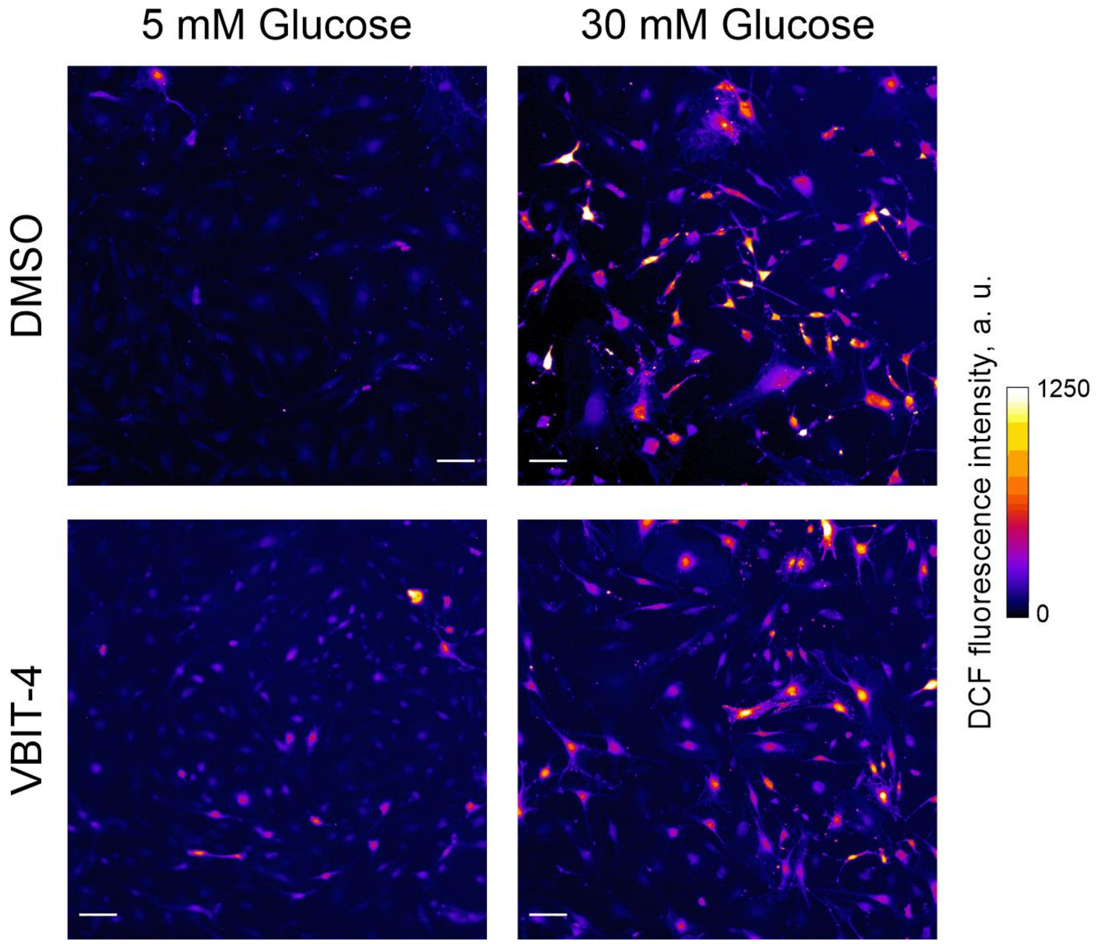

Figure S3. Representative images of the intensity of 2',7'-dichlorodihydrofluorescein diacetate (H2DCFDA) fluorescence signals in the primary culture of mouse microvascular endothelial cells from four experimental groups: 1) 5 mM glucose + 0.1% DMSO; 2) 5 mM glucose + 5 μM VBIT-4; 3) 30 mM glucose + 0.1% DMSO; 4) 30 mM glucose + 5 μM VBIT-4. The scale bar is 100 μm.

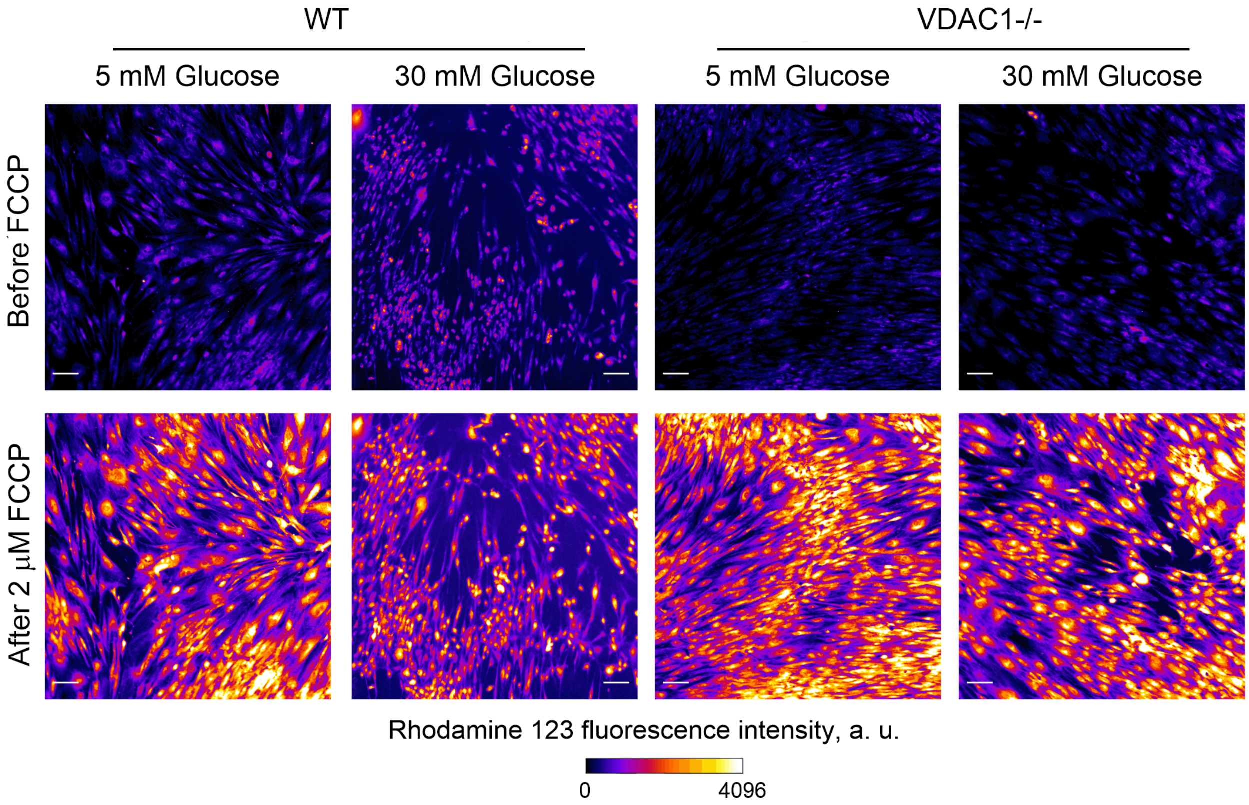

Figure S4. Typical images of the intensity of rhodamine 123 fluorescence signals in the culture of primary human skin fibroblasts with normal (WT) and reduced (VDAC1-/-) expression of VDAC1 under conditions of normo- (5 mM glucose) and hyperglycemia (30 mM glucose) before and after the addition of the uncoupler of oxidative phosphorylation carbonylcyanide-p-trifluoromethoxyphenylhydrazone (FCCP, 2 µm). The scale bar is 100 µm.

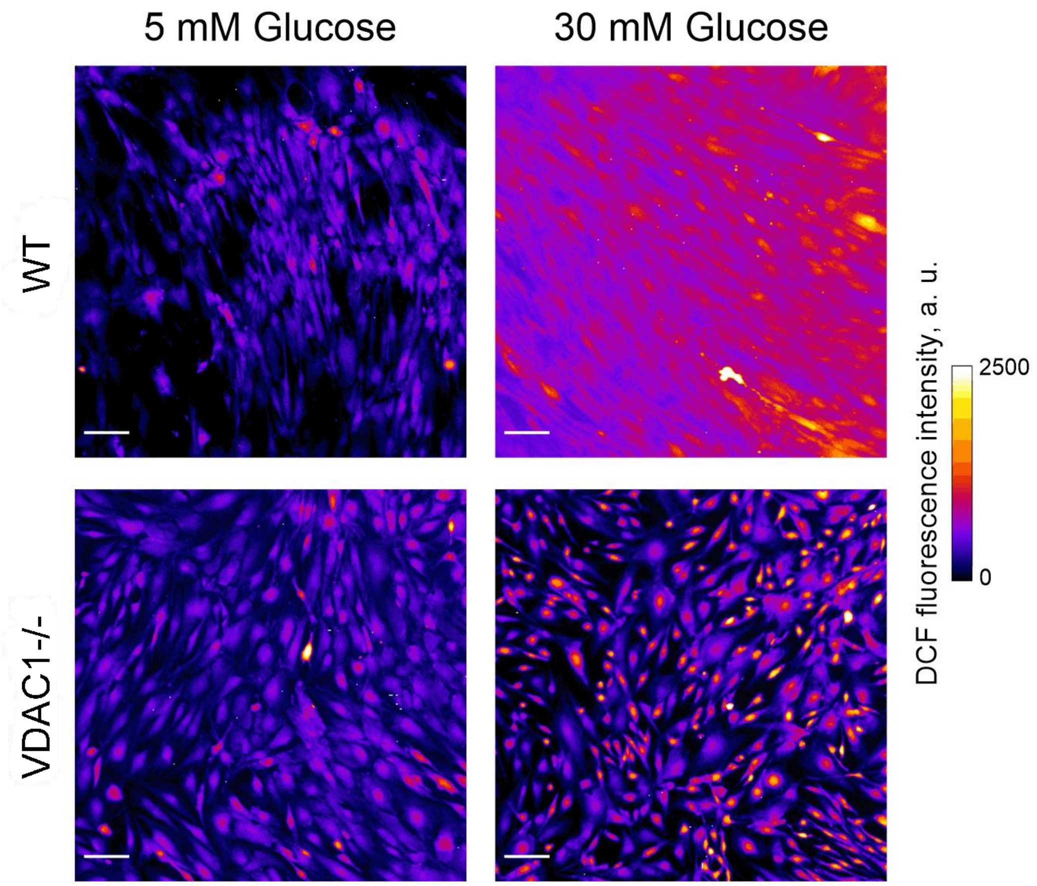

Figure S5. Typical images of the intensity of 2',7'-dichlorodihydrofluorescein diacetate (H2DCFDA) fluorescence signals in the culture of primary human skin fibroblasts with normal (WT) and reduced (VDAC1-/-) expression of VDAC1 under conditions of normo- (5 mM glucose) and hyperglycemia (30 mM glucose). The scale bar is 100 μm.

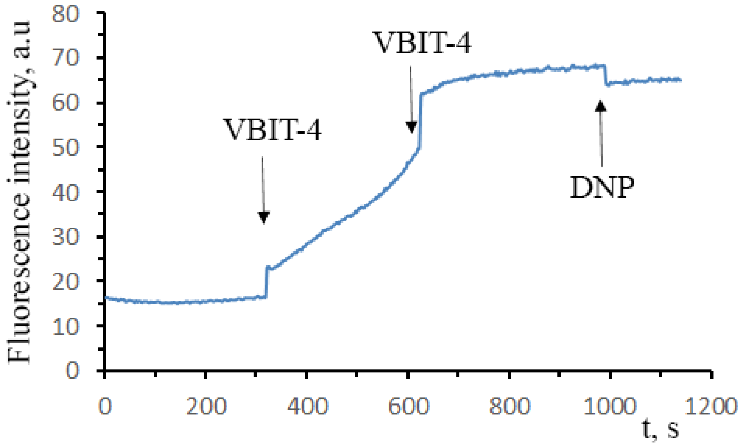

Figure S6. Representative recording of changes in the fluorescence intensity of safranine O, which reveals VBIT-4-induced depolarization of isolated rat liver mitochondria energized by the complex I substrates (2.5 mM potassium glutamate + 2.5 mM malate) Additions: 15 μM VBIT-4, 15 μM VBIT-4, 50 μM DNP. A typical trace is shown (n = 5).
